# Case Image: Cutaneous Larva Migrans Diagnosed via Teledermatology

**DOI:** 10.1002/ccr3.73629

**Published:** 2026-09-23

**Authors:** Vikash Paudel, Deekshanta Sitaula

**Affiliations:** ^1^ Department of Dermatology Patan Academy of Health Sciences Lalitpur Nepal

**Keywords:** cutaneous larva migrans, helminths, parasitic dermatosis, teledermatology

## Abstract

Cutaneous larva migrans is a self‐limiting parasitic dermatosis that typically presents as itchy, serpiginous, migrating skin track and can be diagnosed clinically. This case demonstrates successful diagnosis and treatment through teledermatology, without laboratory or histopathological testing. Recognizing its characteristic appearance is important for prompt diagnosis and effective remote dermatologic care.

## 1 Case Presentation

**FIGURE 1:**
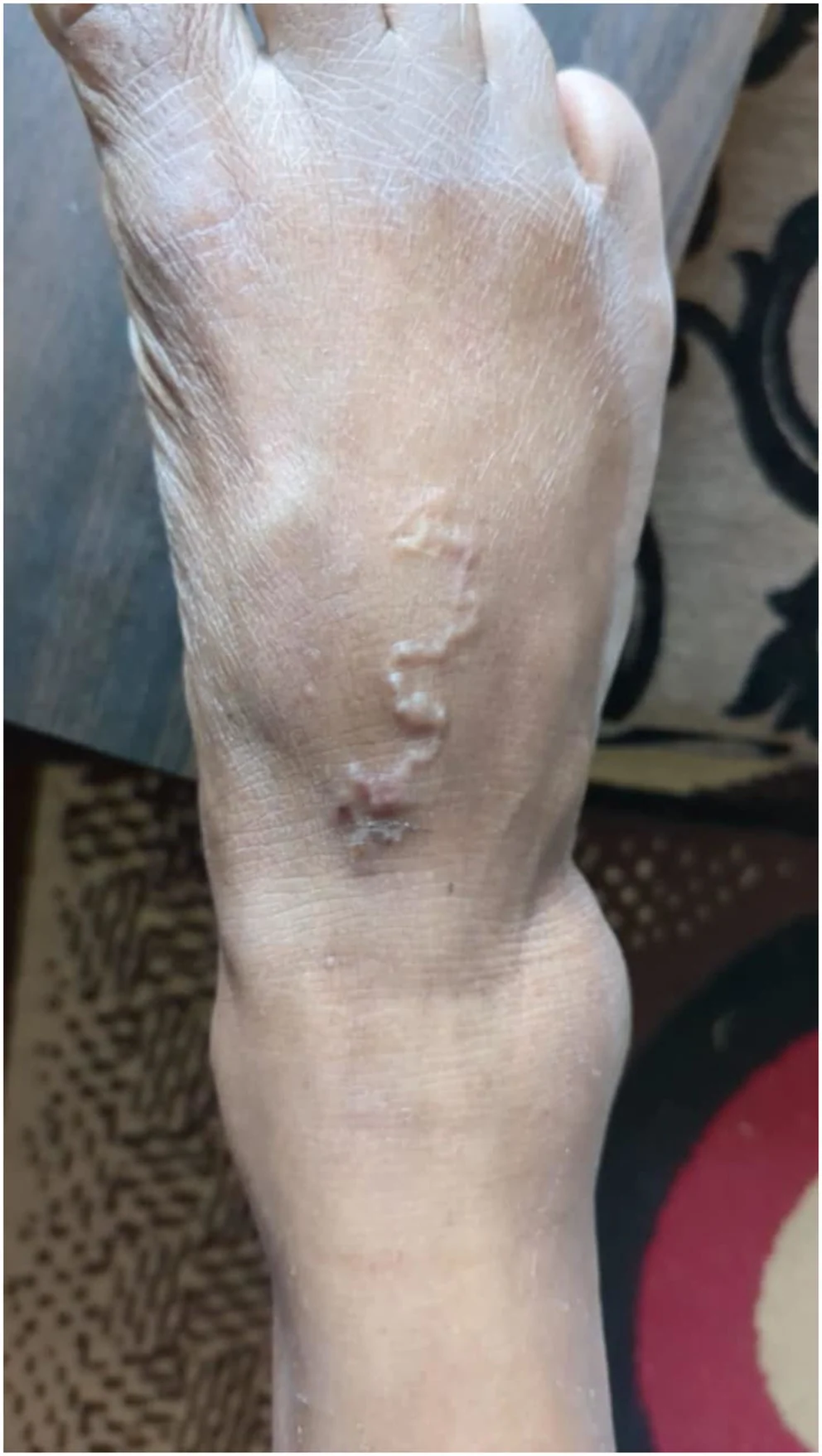
Mild erythematous, serpiginous, thread‐like track over the dorsum of the foot at presentation.

A 58‐year‐old male with no significant past medical history presented via store‐and‐forward teledermatology with a sudden‐onset, intensely pruritic, erythematous, serpiginous, thread‐like lesion over the dorsum of the left foot, which had progressively advanced linearly over several days (Figure 1).

**FIGURE 2:**
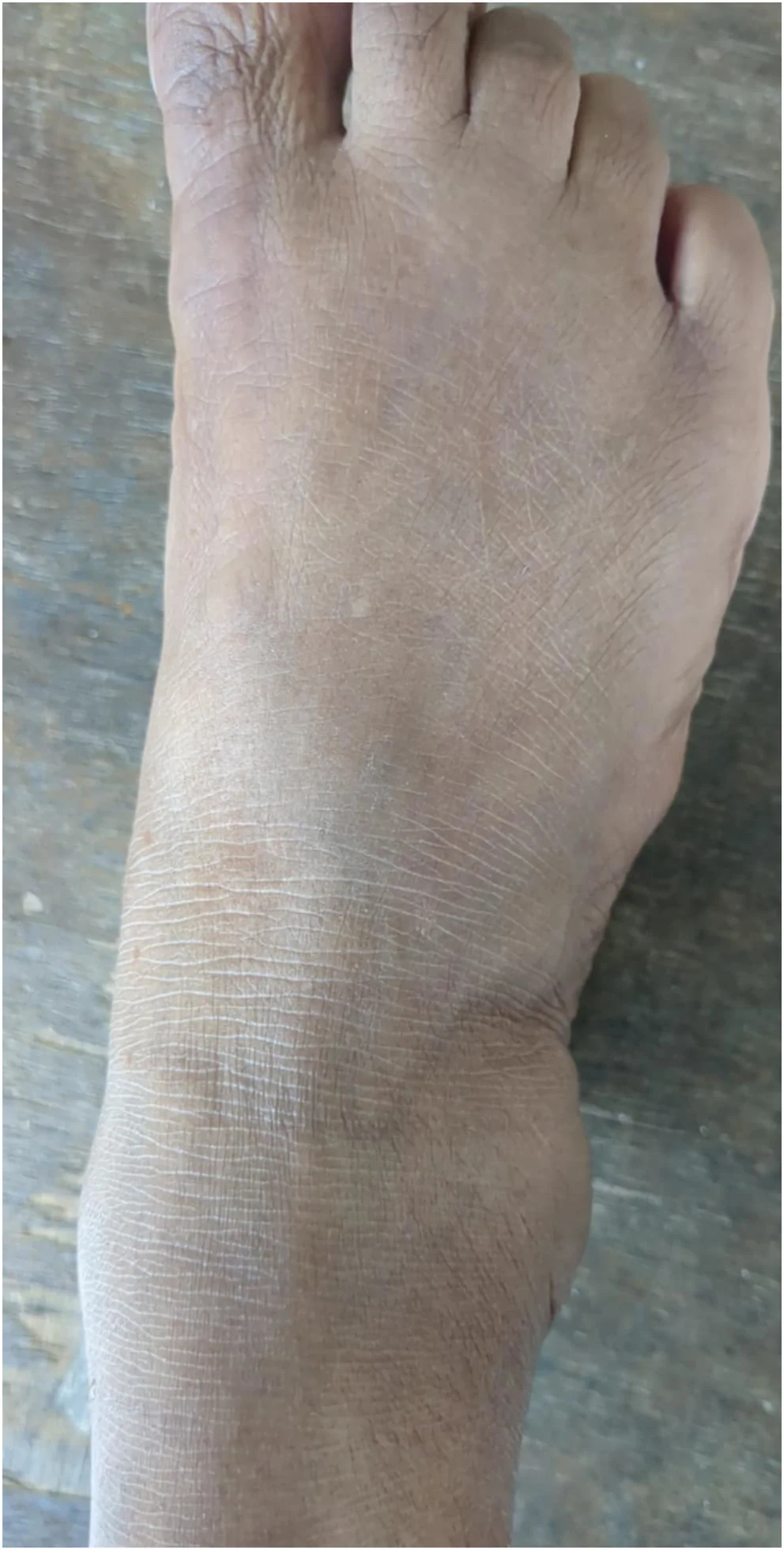
Complete resolution of the lesion following a 3‐day course of oral albendazole (400 mg/day), at 2‐week follow‐up.

There was no associated fever, lymphadenopathy, or systemic symptoms. The patient reported recently walking barefoot on a paddy field. Based on the characteristic clinical morphology and history of environmental exposure, a diagnosis of cutaneous larva migrans (CLM) was made. Differential diagnoses considered included hookworm‐related folliculitis, larva currens, and cutaneous gnathostomiasis. The patient was treated empirically, with complete resolution of itchy lesions at two‐week follow‐up (Figure 2).

## 2 Discussion

The clinical presentation of a solitary, intensely pruritic, erythematous, serpiginous, thread‐like track that rapidly progresses over a few days is typical of cutaneous larva migrans (CLM). The diagnosis was made on teledermatology consultation based on morphology and history alone. Larva currens was considered less likely because the lesion was slowly progressive and persistent on the dorsum of the foot, unlike the rapidly migrating, short‐lived, and typically perianal lesions of larva currens [1]. No laboratory or histopathological investigation was performed, as the presentation was clinically diagnostic. The patient was treated empirically with oral albendazole 400 mg once daily for three days. At the two‐week follow‐up, the pruritus had completely resolved, and the lesion had disappeared, with no recurrence on subsequent follow‐up.

Cutaneous larva migrans (CLM) is a self‐limiting parasitic skin infection caused by animal hookworm larvae, particularly *Ancylostoma braziliense*, that enter the skin through contact with contaminated soil or sand, often in areas exposed to dog or cat feces [1]. Humans are accidental hosts, and the larvae remain confined to the skin. They migrate a few millimeters to centimeters per day, producing a linear, serpiginous track, most commonly on the feet, buttocks, and thighs exposed to contaminated soil [1]. Cutaneous larva migrans is usually diagnosed clinically from the characteristic pruritic, migrating serpiginous track and a history of exposure to contaminated soil or sand. Laboratory tests are generally non‐diagnostic, and biopsy is rarely needed. Oral ivermectin or albendazole is highly effective, with albendazole commonly given at 400 mg daily for three days [2]. Relapsed or refractory cases may need prolonged or combined albendazole and ivermectin therapy [2]. Teledermatology relies primarily on visual assessment of cutaneous morphology supplemented by clinical history. A 2026 systematic review and meta‐analysis of 155 studies found a pooled diagnostic concordance of 76% between teledermatology and in‐person assessment across skin conditions, supporting its use as a reliable alternative in appropriately selected cases [3]. This case illustrates that classic, visually diagnostic dermatoses such as CLM can be safely and effectively managed through teledermatology, particularly relevant in resource‐limited settings where in‐person dermatology access may be delayed.

## Author Contributions

**Vikash Paudel:** conceptualization, supervision, methodology, writing – review and editing. **Deekshanta Sitaula:** conceptualization, investigation, data curation, writing – original draft, writing – review and editing.

## Funding

The authors have nothing to report.

## Consent

The patient provided written informed consent for the publication of their case details, including photographs.

## Conflicts of Interest

The authors declare no conflicts of interest.

## Data Availability

The data that support the findings of this study are available from the corresponding author upon reasonable request.
